# Selective Cytotoxic and Antiproliferative Effects of Extracts from Four Mexican Medicinal Plants in Human Cancer and Non-Cancerous Cell Lines

**DOI:** 10.3390/molecules31030549

**Published:** 2026-02-04

**Authors:** Joel Daniel Castañeda-Espinoza, Yessica Arisbeth Alvarez Soto, Silvia Marquina-Bahena, Guillermo Antonio Madariaga Sosa, Karina Lizbeth Zagal Laguna, Araceli Guerrero-Alonso, Enrique Salas-Vidal, Janette Furuzawa-Carballeda, Juan M. Uriostegui-Velarde, Carlos Mojica Cardoso, Abraham Noé Anzurez Jiménez, Estela Carranza Valencia, Erick Ayala Calvillo, Jessica Nayelli Sánchez-Carranza

**Affiliations:** 1 Centro de Desarrollo de Productos Bióticos, Instituto Politécnico Nacional, Carretera Yautepec—Jojutla s/n-km. 85, San Isidro, Yautepec 62739, Morelos, Mexico; daniel_rojo8@hotmail.com; 2 Facultad de Farmacia, Universidad Autónoma del Estado de Morelos, Av. Universidad 1001, Col. Chamilpa, Cuernavaca 62209, Morelos, Mexico; arisbetha600@gmail.com (Y.A.A.S.); guillermo.madariaga@farmacia.uaem.edu.mx (G.A.M.S.); karii.zagal@gmail.com (K.L.Z.L.); eac_ff@uaem.mx (E.A.C.); 3 Centro de Investigaciones Químicas-IICBA, Universidad Autónoma del Estado de Morelos, Av. Universidad 1001, Col. Chamilpa, Cuernavaca 62209, Morelos, Mexico; smarquina@uaem.mx (S.M.-B.); q.aguerreroa@gmail.com (A.G.-A.); 4 Departamento de Genética del Desarrollo y Fisiología Molecular, Instituto de Biotecnología, Universidad Nacional Autónoma de Mexico, Cuernavaca 62209, Morelos, Mexico; enrique.salas@ibt.unam.mx; 5 Departamento de Cirugía Experimental, Instituto Nacional de Ciencias Médicas y Nutrición Salvador Zubirán, Ciudad de Mexico 14080, Mexico; jfuruzawa@gmail.com; 6 Escuela de Medicina, Universidad Panamericana, Ciudad de Mexico 03920, Mexico; 7 Facultad de Ciencias Biológicas, Universidad Autónoma del Estado de Morelos, Av. Universidad 1001, Col. Chamilpa, Cuernavaca 62209, Morelos, Mexico; juan.uriosteguive@docentes.uaem.edu.mx; 8 Laboratorio de Patología, Hospital del Niño Morelense, Av. de la Salud #1 Col. Benito Juárez, Emiliano Zapata 62765, Morelos, Mexico; carlos.mojica.cardoso@gmail.com (C.M.C.); abrajam.anzurez@gmail.com (A.N.A.J.); 9 Instituto Politécnico Nacional, Av. Luis Enrique Erro S/N, Unidad Profesional Adolfo López Mateos, Zacatenco, Alcaldía Gustavo A. Madero, Ciudad de Mexico 07738, Mexico; ecarranza@ipn.mx

**Keywords:** Mexican medicinal plants, selective cytotoxicity, antiproliferative activity, *Semialarium mexicanum*, *Eryngium heterophyllum*, *Cochlospermum vitifolium*, *Piper auritum*, cervical cancer, HeLa, SiHa, human uterine fibroblasts (HUF), *IC*_50_, selectivity index

## Abstract

**Background**: Medicinal plants used in traditional Mexican medicine represent a valuable source of bioactive compounds with potential anticancer activity. Beyond cytotoxic potency, selectivity toward cancer cells over normal cells is a critical toxicological parameter for identifying safer therapeutic candidates. This study aimed to evaluate the selective cytotoxic and antiproliferative effects of extracts from four Mexican medicinal plants across human cancerous and non-cancerous cell lines. **Methods**: Hexane, acetone, and methanolic extracts from *Semialarium mexicanum*, *Eryngium heterophyllum*, *Piper auritum*, and *Cochlospermum vitifolium* were evaluated in a panel of human cancer cell lines and non-tumoral models, including primary human uterine fibroblasts (HUFs). Cytotoxicity was assessed after 48 h of treatment using increasing extract concentrations, and selectivity indices were calculated. Cell cycle distribution and nuclear morphology analyses were performed to explore antiproliferative effects. Additionally, GC–MS-based chemical profiling was conducted on selected extracts to obtain a tentative characterization of major bioactive constituents. **Results**: The extracts exhibited differential cytotoxic profiles depending on plant species and solvent polarity. The hexane extract of *Semialarium mexicanum* showed the highest cytotoxic potency and selectivity toward cervical cancer cells, with half-maximal inhibitory concentration (*IC*_50_); values of 15.9 ± 1.8 µg/mL and 17.2 ± 2.8 µg/mL in HeLa and SiHa cells, respectively, and selectivity index (SI) values > 5 when compared with primary human uterine fibroblasts (HUF). Extracts of *Eryngium heterophyllum* displayed moderate cytotoxic activity (*IC*_50_ = 20–30 µg/mL in HeLa cells) with intermediate selectivity, whereas *Cochlospermum vitifolium* showed solvent-dependent effects and *Piper auritum* exhibited limited cytotoxicity. Cell cycle analysis revealed an increased sub-G1 population, and nuclear morphology assays demonstrated chromatin condensation and fragmentation in cancer cells, supporting an antiproliferative mechanism. GC–MS analysis of the hexane extract of *Semialarium mexicanum* suggested the presence of triterpenoid-related and other lipophilic compounds potentially associated with its selective anticancer activity. **Conclusions**: These findings provide in vitro evidence of selective anticancer activity of Mexican medicinal plant extracts and establish a basis for future mechanistic studies medicinal plant extracts and lay the groundwork for future mechanistic investigations.

## 1. Introduction

Traditional Mexican Medicine (TMM) represents an ancestral therapeutic system deeply rooted in the indigenous, mestizo, and rural cultures of Mexico. This medical tradition is primarily based on the empirical use of medicinal plants, often integrated with ritual and cultural practices, and has been formally recognized by the World Health Organization as a fundamental component of biocultural heritage and a valuable source of bioactive compounds with pharmacological potential [1,2,3,4,5].

Mexico is considered one of the world’s most important reservoirs of medicinal plant diversity. To date, more than 4000 plant species with documented medicinal use have been reported, of which at least 300 are traditionally employed to treat conditions associated with inflammation, tumors, “lumps,” cancer-like disorders, and hematological or reproductive diseases [1,2,3,4]. Several genera, including *Semialarium mexicanum* (syn. *Hippocratea excelsa*), *Eryngium heterophyllum*, *Cochlospermum vitifolium* and *Piper auritum*, are widely used in Mexican traditional medicine for the treatment of inflammatory disorders, infections, gastrointestinal ailments, and conditions associated with abnormal cell growth. Previous studies on species belonging to these genera have reported a broad range of bioactivities, such as antioxidant, anti-inflammatory, antimicrobial, and cytotoxic effects, suggesting their potential relevance in cancer research. However, despite their ethnopharmacological importance, many Mexican medicinal plants remain insufficiently characterized under controlled experimental conditions, particularly regarding their selective anticancer activity [1,2,3,4,5,6,7,8].

Despite their extensive traditional use, systematic scientific evidence supporting the cytotoxic and antiproliferative activities of many Mexican medicinal plants remains limited. This gap has driven a growing interest in their experimental evaluation using in vitro cancer models, aimed at identifying extracts or metabolites with potential antitumoral properties [6]. The renewed global interest in natural products over recent decades is largely attributable to their remarkable chemical diversity, particularly their richness in secondary metabolites such as terpenoids, flavonoids, alkaloids, lignans, and quinones, have also demonstrated the ability to interfere with DNA replication, disrupt redox homeostasis, and activate programmed cell death. These mechanisms provide a strong biological basis for evaluating plant extracts rich in these compound classes as potential anticancer agents [9]. Notably, it has been estimated that approximately 60% of currently approved anticancer drugs are derived directly or indirectly from natural products, such as paclitaxel, vincristine, and camptothecin derivatives, originating from plant sources. In addition, numerous preclinical studies have demonstrated that crude plant extracts and isolated compounds can exhibit selective cytotoxicity toward cancer cells while sparing normal cells. These findings highlight the importance of systematically exploring medicinal plants as sources of bioactive compounds with potential therapeutic value [9].

In this context, the aim of the present study was to systematically evaluate four Mexican medicinal plants—*Semialarium mexicanum* (*S. mexicanum*), *Eryngium heterophyllum* (*E. heterophyllum*), *Cochlospermum vitifolium* (*C. vitifolium*), and *Piper auritum* (*P. auritum*)—selected based on their documented traditional use and ethnopharmacological relevance. The specific objectives were to identify plant extracts with high cytotoxic potency and selectivity, to compare their biological effects across different cancer and non-cancer cell lines, and to generate experimental evidence supporting their potential as sources of anticancer agents.

## 2. Results

### 2.1. Plant Material

**Figure 1:**
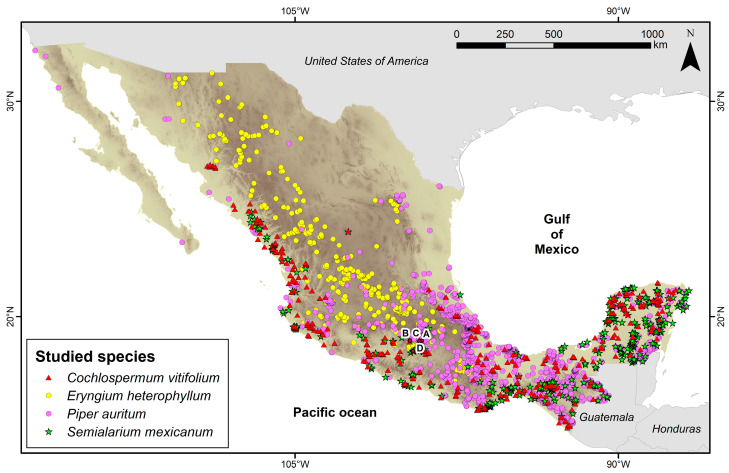
Occurrence records of the four species in Mexico. Letter A indicates the collection site of *C. vitifolium*, B of *E. heterophyllum*, C of *S. mexicanum*, and D of *P. auritum*. For Mexico, the background color intensity represents elevation (m a.s.l.): light tones indicate low elevation, and dark tones indicate high elevation.

The four species are widely distributed in Mexico. According to the Global Biodiversity Information Facility (GBIF; Figure 1) [10], *C. vitifolium* has 414 occurrence records in the country (GBIFa), *E. heterophyllum* 428 (GBIFb), *P. auritum* 1824 (GBIFc), and *S. mexicanum* 542 (GBIFd) [11,12,13,14]. The specimens were collected in rural and semi-urban areas of the states of Morelos, Guerrero, and the State of Mexico (Figure 1). *C. vitifolium* was collected in Temixco, Morelos (18.8606 N, 99.2319 W); *E. heterophyllum* in Ixtapan de la Sal, State of Mexico (18.8903 N, 99.6733 W); *P. auritum* in Yautepec, Morelos (18.8917 N, 99.1208 W); and *S. mexicanum* in Iguala, Guerrero (18.4031 N, 99.5331 W; Figure 1).

The taxonomic identification was carried out by the specialist, Master of Science Gabriel Flores Franco, and a representative sample of each species was deposited in the Herbarium of the Autonomous University of the State of Morelos (HUMO-UAEM), where the corresponding registration codes were assigned, as shown in Table 1.

#### Plant Material Extraction

Twelve extracts were obtained from the plants *C. vitifolium*, *E. heterophyllum*, *P. auritum* and *S. mexicanum*, organic solvents with ascending polarity were used (hexane, acetone, and methanol). The extracts were prepared from 150 g of ground plant material. The yields obtained from the extracts are shown in Table 2.

The methanolic and acetonic extract of *P. auritum* had the highest yield at 16.2% and 16.02% respectively, followed by the methanolic extract of *E. heterophyllum* (9.81%). On the other hand, the hexane extract of *C. vitifolium* showed the lowest yield, followed by *S. mexicanum* (0.49% and 1.14%, respectively).

### 2.2. Biological Activity of the Extracts

#### 2.2.1. Effect of Mexican Medicinal Plant Extracts on Cell Viability in Human Cancer and Normal Cell Lines

To evaluate the cytotoxic effects of the hexane, acetone, and methanol extracts of *C. vitifolium*, *E. heterophyllum*, *P. auritum*, and *S. mexicanum*—selected medicinal plants from Mexican Traditional Medicine—their impact on cell viability was assessed across a panel of human cancer cell lines representing different tissue origins, including cervical cancer (HeLa and SiHa), prostate cancer (PC-3), breast cancer (MCF-7), and lung cancer (H1299).

In parallel, cytotoxicity was evaluated in non-cancerous human cell models, including normal human dermal fibroblasts (HFF-1) and primary uterine fibroblast cells (HUF), as well as immortalized but non-tumorigenic cell lines such as human keratinocytes (HaCaT) and human embryonic kidney cells (HEK-293), in order to assess the selectivity of the extracts toward malignant cells.

**Figure 2:**
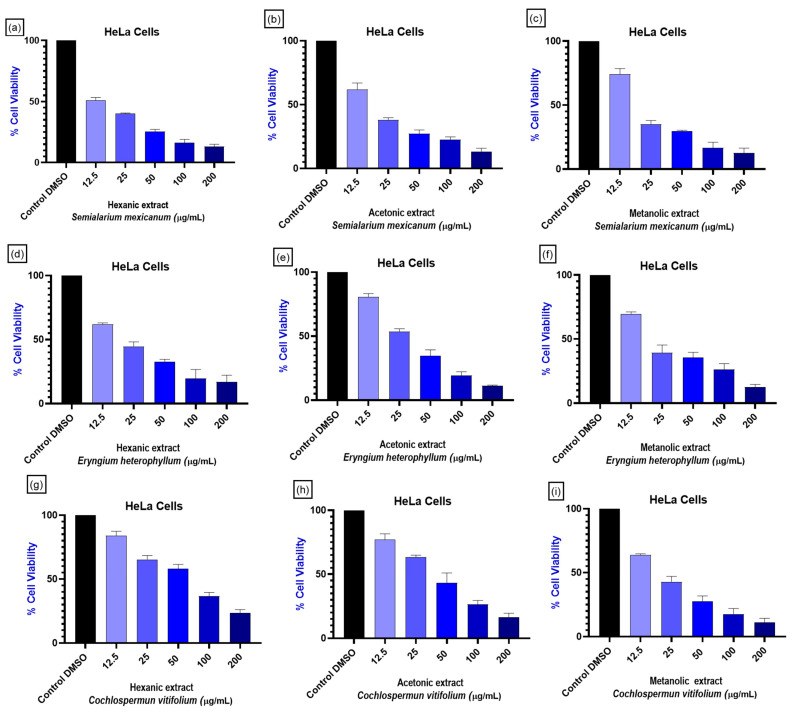
Effect on HeLa cell viability of plant extracts. HeLa cells were treated with hexane, acetone, and methanol extracts from *Semialarium mexicanum* (**a**–**c**), *Eryngium heterophyllum* (**d**–**f**), and *Cochlospermum vitifolium* (**g**–**i**) at concentrations ranging from 0 to 200 µg/mL. Cell viability is expressed as percentage of the negative control (0.1% DMSO). All experiments were performed in triplicate, and data show a concentration-dependent decrease in cell viability, with extract- and species-dependent differences in potency.

Given the large number of treatments generated by the use of three extraction solvents per plant species and the multiple cell lines analyzed, representative concentration–response curves were selected based on cytotoxic potency, selectivity toward cancer cells, and consistency of effects across different tumor types. As illustrated in Figure 2, the hexane, acetone, and methanol extracts of *S. mexicanum* and *E. heterophyllum*, as well as the methanolic extract of *C. vitifolium*, produced a clear concentration-dependent decrease in cell viability in cancer cell lines derived from cervix, breast, lung, and prostate tissues. Notably, this effect was most pronounced in cervical cancer cells, particularly in the HeLa cell line, indicating a higher sensitivity of these cells to the evaluated extracts.

**Figure 3:**
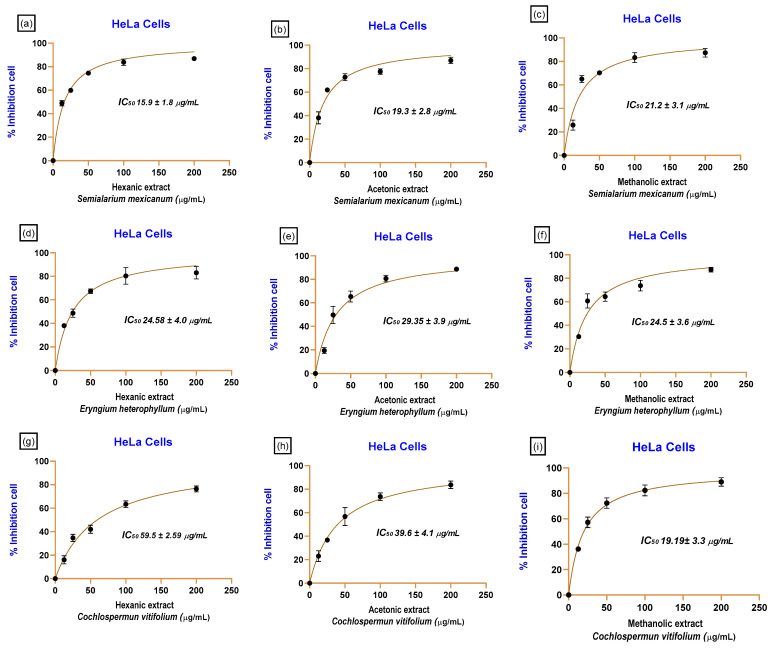
Concentration–response curves showing proliferation inhibition in HeLa cells treated with plant extracts. Percentage of proliferation inhibition in HeLa cells treated with hexane, acetone, and methanolic extracts of *Semialarium mexicanum* (**a**–**c**), *Eryngium heterophyllum* (**d**–**f**), and *Cochlospermum vitifolium* (**g**–**i**) at concentrations ranging from 0 to 200 µg/mL. Curves were used to calculate *IC*_50_ values. Cells treated with 0.1% DMSO served as the negative control. Data are expressed as mean ± SD of three independent experiments performed in triplicate.

Subsequently, based on the initial evaluation of extract effects on cell viability, concentration–response curves were generated and expressed as the percentage of proliferation inhibition as a function of extract concentration (Figure 3). These curves were used to quantitatively determine the half-maximal inhibitory concentration (*IC*_50_ defined as the concentration of a compound or extract required to inhibit 50% of cellular proliferation or viability relative to untreated control cells under identical experimental conditions. *IC*_50_ values provide a robust and comparative measure of biological potency, enabling the assessment of cytotoxic and antiproliferative effects of different extracts across multiple cell lines.

All extract treatments were systematically analyzed, and the resulting cytotoxicity data are summarized as *IC*_50_ values in Table 3, reported as mean ± SD from three independent experiments performed in triplicate.

Overall, the extracts exhibited cytotoxic effects that depended on both the extraction solvent and the cancer cell line, revealing distinct and tissue-specific responses. The lowest *IC*_50_ values were predominantly observed in cervical cancer cell lines (HeLa and SiHa). For *S. mexicanum*, the hexane extract showed the highest cytotoxic potency, with *IC*_50_ values of 15.9 ± 1.8 µg/mL in HeLa cells and 17.16 ± 2.8 µg/mL in SiHa cells. In the case of *E. heterophyllum*, cytotoxic activity was observed, with *IC*_50_ values of 24.58 ± 4.0 µg/mL (HeLa) and 20.4 ± 2.4 µg/mL (SiHa) for the hexane extract. Additionally, the methanolic extract of *C. vitifolium* exhibited notable activity in cervical cancer cells, with *IC*_50_ values of 19.19 ± 3.3 µg/mL in HeLa cells and 20.45 ± 2.5 µg/mL in SiHa cells. In contrast, *P. auritum* showed low cytotoxicity, as reflected by the methanolic extract, which displayed *IC*_50_ values of 50.7 ± 5.1 µg/mL in HeLa cells and 75.5 ± 5.1 µg/mL in SiHa cells. Paclitaxel, used as a positive control, consistently exhibited low *IC*_50_ values, confirming the robustness and reliability of the experimental model.

To assess the selectivity of the evaluated extracts, their effects were further analyzed in a panel of non-cancerous or low malignant potential cell models, enabling a biologically relevant comparison across normal and immortalized cells from different tissue origins. This panel included normal human dermal fibroblasts (HFF-1), widely used as a reference model of normal stromal cells; spontaneously immortalized human keratinocytes (HaCaT), which represent a non-tumorigenic epithelial model [15]; and human embryonic kidney epithelial cells (HEK293), an immortalized but non-tumorigenic cell line originally generated by adenoviral transformation [16,17].

In addition, to provide a more tissue-specific comparison relevant to cervical cancer, the extracts were also evaluated in HUF cells, corresponding to primary normal cervical fibroblasts, which serve as a physiologically relevant non-cancerous model of cervical origin [18].

Notably, all non-cancerous cell lines consistently exhibited higher *IC*_50_ values than those observed in cancer cell lines, indicating reduced sensitivity to the evaluated extracts. Among the non-cancerous models, HUF and HFF-1 cells were the least sensitive, followed by HaCaT and HEK293 cells (Table 4).

**Figure 4:**
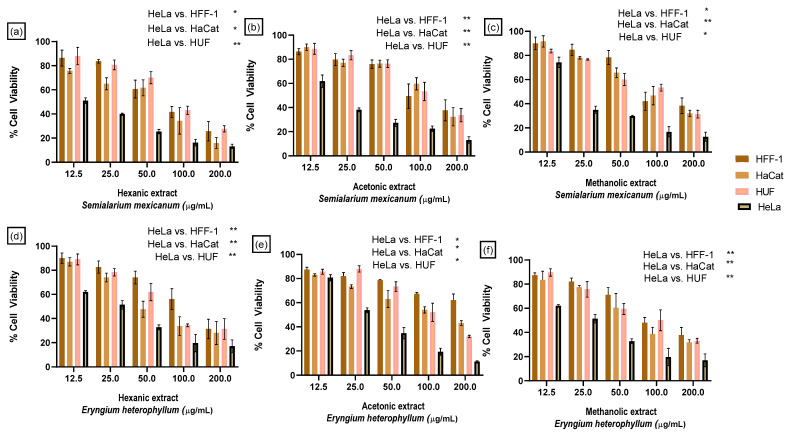
Comparative cytotoxic effects of selected Mexican medicinal plant extracts across cancerous and non-cancerous cell lines. Bar graphs depict the percentage of cell viability inhibition induced by hexane, acetone, and methanolic extracts of *Semialarium mexicanum* and *Eryngium heterophyllum* in human cervical cancer cell lines HeLa and non-cancerous cell models, including HFF-1, HUF, HaCaT, and HEK293 cells. Data are expressed as mean ± SD. Statistical analysis was performed using one-way ANOVA followed by Dunnett’s multiple comparisons test, using HeLa cells as the reference group. Asterisks indicate statistically significant differences relative to HeLa cell (* *p* < 0.05) and (** *p* < 0.05).verall, non-cancerous cells exhibited lower sensitivity to the extracts compared to cervical cancer cells. Among them, primary uterine fibroblast cells (HUF) and fibroblasts (HFF-1) were the least affected.

Figure 4 presents a comparative analysis of the effects of the hexanic, acetonic, and methanolic extracts of *S. mexicanum* and *E. heterophyllum*, which were previously identified as the most active species based on their cytotoxic responses in the cervical cancer cell lines HeLa and SiHa.

Notably, HeLa cells remained the most sensitive to all treatments, confirming a preferential cytotoxic effect toward cervical cancer cells. This response pattern indicates a selective cytotoxic profile of the evaluated extracts, characterized by enhanced activity against cancer cells while maintaining a favorable safety margin in normal and immortalized cell lines.

**Figure 5:**
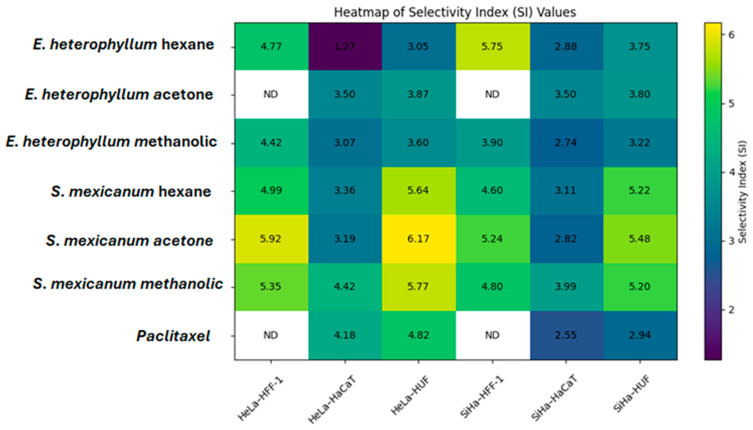
Heatmap of SI values selected plant extracts. Heatmap representation of SI values calculated for hexane, acetone, and methanolic extracts of *Eryngium heterophyllum* and *Semialarium mexicanum* in cervical cancer cell lines (HeLa and SiHa) relative to non-cancerous cell models (HFF-1, HaCaT, and HUF). SI values were calculated as the ratio between *IC*_50_ values in non-cancerous cells and the corresponding *IC*_50_ values in cancer cells. Higher SI values indicate greater selectivity toward cancer cells. Paclitaxel was included as a reference compound. ND indicates not determined.

This selectivity is further supported by the selectivity index (SI) values summarized in Table 5. To facilitate a comparative visualization of the selectivity profile, a heatmap Figure 5 representation of the SI was generated using the values summarized in Table 6. The SI was calculated as the ratio between the *IC*_50_ obtained in non-cancerous cell lines (HFF-1, HaCaT, and HUF) and the *IC*_50_ determined in cervical cancer cell lines (HeLa and SiHa), where higher SI values indicate greater selectivity toward cancer cells.

The hexanic, acetonic, and methanolic extracts of *S. mexicanum* show SI > 5 against at least one HeLa cell line, indicating high selectivity for tumor cells over HFF-1 and HUF. In comparison, *E. heterophyllum* exhibits moderate SI values (3–4.5).

HEK293 cells are adenovirus-transformed, immortalized embryonic kidney cells and therefore do not represent non-transformed normal tissue; accordingly, they were excluded from Selectivity Index calculations and used exclusively as a complementary non-tumoral sensitivity model to provide additional information on extract cytotoxicity in a rapidly proliferating cell system.

#### 2.2.2. Morphological Assessment of Extract-Induced Cytotoxicity in Cancer and Normal Cells

To complement the quantitative data obtained from cell viability, *IC*_50_ determination, and proliferation inhibition assays, the morphological effects induced by the most potent extracts were evaluated by optical microscopy. A concentration of 20 µg/mL—approximately corresponding to the *IC*_50_ values observed in HeLa and SiHa cells—was selected for all treatments, including normal HUFs, to allow direct comparison.

**Figure 6:**
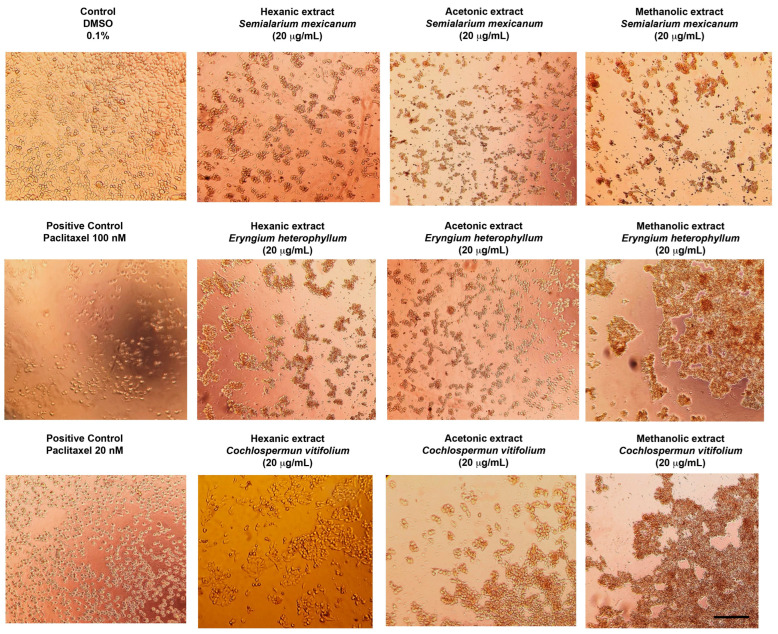
Representative micrographs of HeLa cells treated for 48 h with hexanic, acetonic, and methanolic extracts of *Semialarium mexicanum*, *Eryngium heterophyllum*, and *Cochlospermum vitifolium* (20 µg/mL), compared to vehicle control (0.1% DMSO) and positive control paclitaxel (20 and 100 nM). The images reveal a marked reduction in cell density, morphological changes indicative of cell death, and the formation of aggregates following extract exposure. Images were acquired at 10× magnification. Scale bar 100 µm.

**Figure 7:**
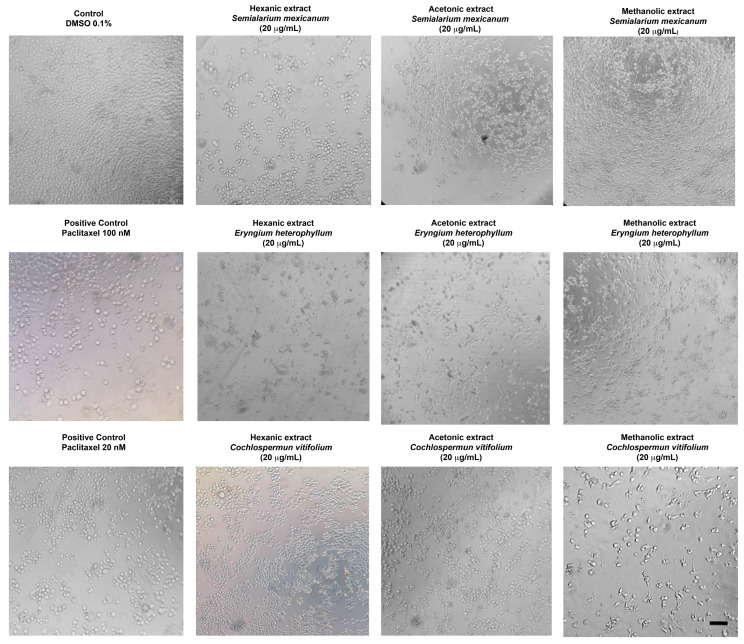
Representative phase-contrast micrographs of SiHa cells after 48 h of treatment with hexanic, acetonic, and methanolic extracts of *Semialarium mexicanum*, *Eryngium heterophyllum*, and *Cochlospermum vitifolium* (20 µg/mL), compared to vehicle control (0.1% DMSO) and positive control paclitaxel (20 and 100 nM). The images show reduced confluency, cell detachment, and morphological changes indicative of extract-induced cell death. Images were acquired at 10× magnification. Scale bar 100 µm.

**Figure 8:**
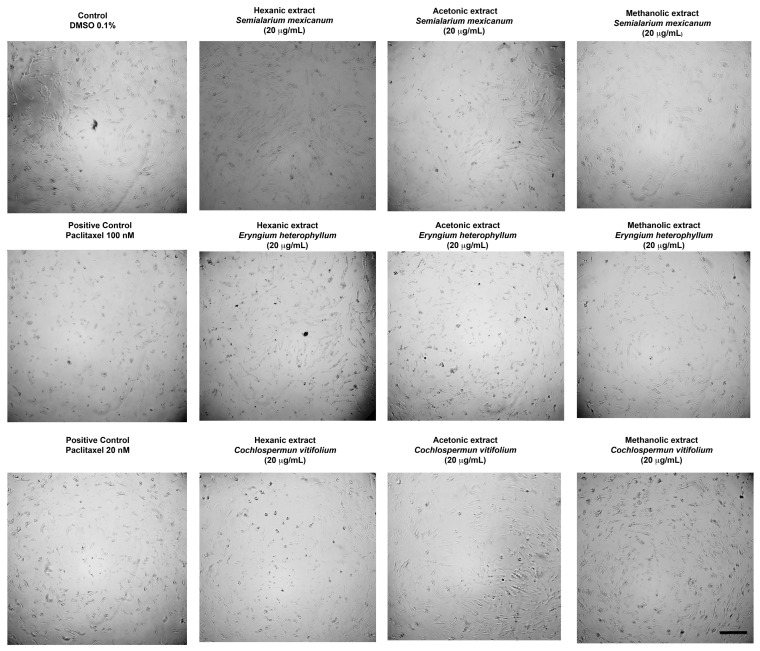
Representative phase-contrast micrographs of HUF cells after 48 h of treatment with hexanic, acetonic, and methanolic extracts of *Semialarium mexicanum*, *Eryngium heterophyllum*, and *Cochlospermum vitifolium* (20 µg/mL), compared to vehicle control (0.1% DMSO) and positive control paclitaxel (20 and 100 nM). At this concentration, no significant morphological changes were observed in HUF cells, supporting the selective cytotoxicity of the extracts toward cancer cells. Images were acquired at 10× magnification. Scale bar 100 µm.

As shown in Figure 6 (HeLa cells) and Figure 7 (SiHa cells), treatment with the selected extracts resulted in evident morphological alterations characteristic of cytotoxic damage, including cell rounding, loss of adherence, and reduced cell density when compared with untreated controls. In contrast, HUFs exposed to the same concentration (Figure 8) displayed preserved morphology and cell organization, with no apparent structural damage relative to control cells.

Overall, these microscopy observations are consistent with the quantitative cytotoxicity data and clearly illustrate the selective cytotoxic effects of the extracts toward cancer cells, while maintaining the morphological integrity of non-cancerous cells.

#### 2.2.3. Effects of Hexanic, Acetonic, and Methanolic Extracts (*Eryngium*, *Semialarium* and *Cochlospermum*), on Cell Cycle Progression and Nuclear Morphology in HeLa Cells

**Figure 9:**
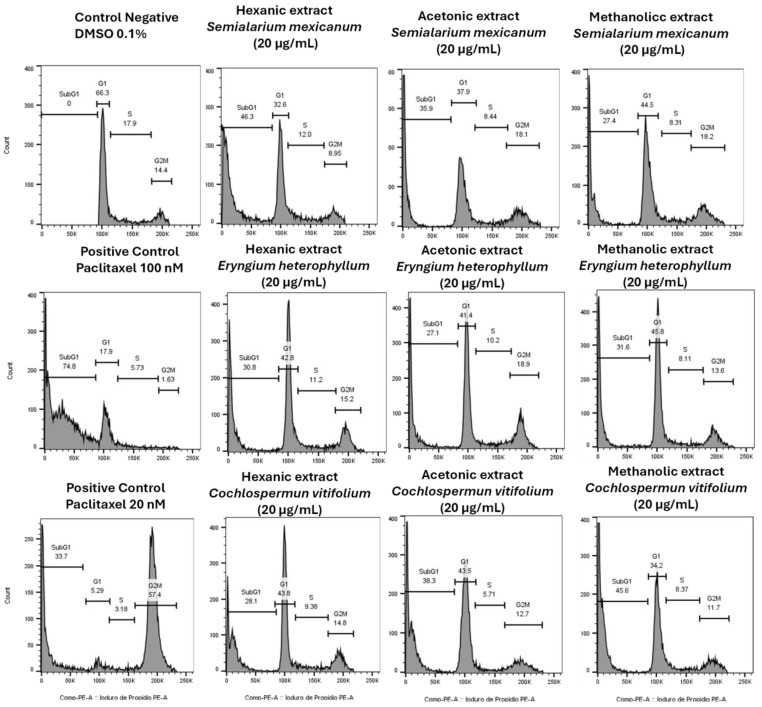
Cell cycle analysis by flow cytometry of cells treated with extracts from *Semialarium mexicanum*, *Eryngium heterophyllum*, and *Cochlospermum vitifolium* in HeLa cells. Representative histograms show the DNA content distribution (propidium iodide staining) following treatment with hexanic, acetonic, and methanolic extracts 20 µg/mL. Negative control (0.1% DMSO) displaying a standard distribution of G1, S, and G2/M phases. Positive controls with Paclitaxel (100 nM and 20 nM) showing apoptosis induction (increased SubG1 sub-population) and G2/M phase arrest.

While the primary aim of this study was to evaluate extract selectivity toward normal cells and identify those with a safe profile, we further explored their impact on cancer cell proliferation. Cell cycle analysis in HeLa cells (Figure 9) revealed that all extracts induced an increase in the sub-G1 population, indicative of DNA fragmentation and suggestive of cell death, with the hexanic extract of *S. mexicanum* producing the most pronounced effect, thereby corroborating the cytotoxicity and selectivity observed in previous assays.

**Figure 10:**
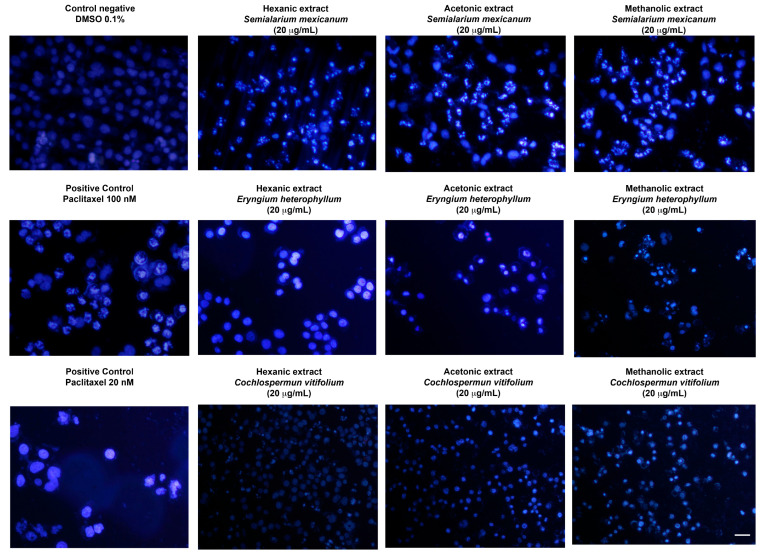
Nuclear morphology of HeLa cells assessed by DAPI staining. Representative fluorescence micrographs (10× magnification) of cells treated for 24 h with hexanic, acetonic, and methanolic extracts (20 µg/mL) of *Semialarium mexicanum*, *Eryngium heterophyllum*, and *Cochlospermum vitifolium* in HeLa cells. Negative controls (0.1% DMSO) display intact nuclei with uniform chromatin, whereas positive controls (paclitaxel 20 and 100 nM) and extract-treated cells exhibit apoptotic features, including chromatin condensation, nuclear fragmentation, and brightly fluorescent apoptotic bodies. Scale bar 50 µm.

To complement the evaluation of extract selectivity toward normal cells, we examined their effects on HeLa cell proliferation and nuclear morphology. Cell cycle analysis (Figure 9) revealed that all extracts induced an increase in the sub-G1 population, indicative of DNA fragmentation and suggestive of cell death, with the hexanic extract of *S. mexicanum* producing the most pronounced effect, corroborating previous cytotoxicity results. Consistently, DAPI staining (Figure 10) showed clear nuclear alterations: control cells (0.1% DMSO) displayed intact, uniformly stained nuclei, whereas positive controls (paclitaxel 20 and 100 nM) exhibited classic apoptotic features, including nuclear fragmentation and condensed apoptotic bodies. Treatment with 20 µg/mL of *S. mexicanum, E. heterophyllum*, and *C. vitifolium* induced varying degrees of chromatin condensation, nuclear fragmentation, and pyknotic nuclei, supporting the conclusion that the antiproliferative effects of these extracts could involve programmed cell death.

### 2.3. Chemical Profiling of the Extract

Following the evaluation of cytotoxic and antiproliferative activity, a stepwise chemical profiling approach was applied to gain insight into the main classes of compounds potentially associated with the observed biological effects. The analytical strategy was designed to progress from a general qualitative assessment to more targeted analyses, taking into account both the polarity of the extracts and their biological relevance.

An initial qualitative screening by thin-layer chromatography (TLC) was conducted on the hexane and acetone extracts to obtain a comparative overview of their chemical profiles. Subsequently, the total phenolic content was quantified in the acetone and methanol extracts, given the well-recognized contribution of phenolic compounds to antiproliferative and cytotoxic effects in cancer cells. Finally, due to its higher cytotoxic potency, the hexane extract of *S. mexicanum* was selected for GC–MS analysis to tentatively identify its volatile and semi-volatile constituents.

#### 2.3.1. Thin-Layer Chromatography (TLC) Analysis of Hexane and Acetone Extracts

**Figure 11:**
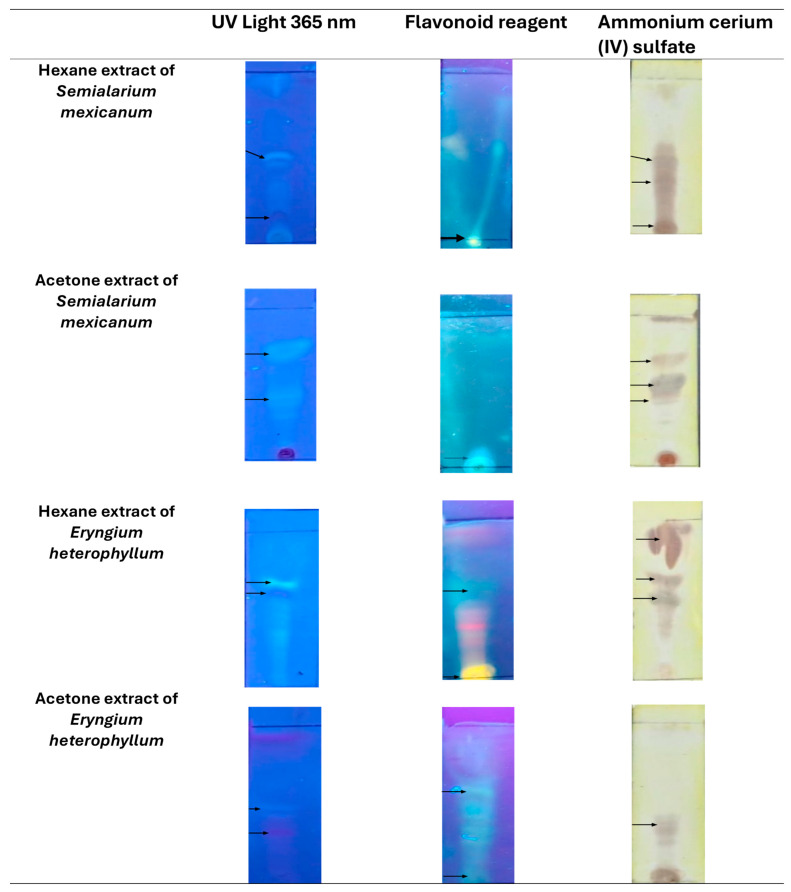
TLC visualization of hexane and acetone extracts of *Semialarium mexicanum* and *Eryngium heterophyllum*. Chromatograms were observed under UV light at 365 nm and after derivatization with a flavonoid-specific reagent and ammonium cerium(IV) sulfate. The arrows indicate the fluorescent bands from orange-yellow to greenish-white, which suggested compounds similar to flavonoids, while dark brown bands after derivatization were consistent with the presence of terpenoid-type constituents.

**Figure 12:**
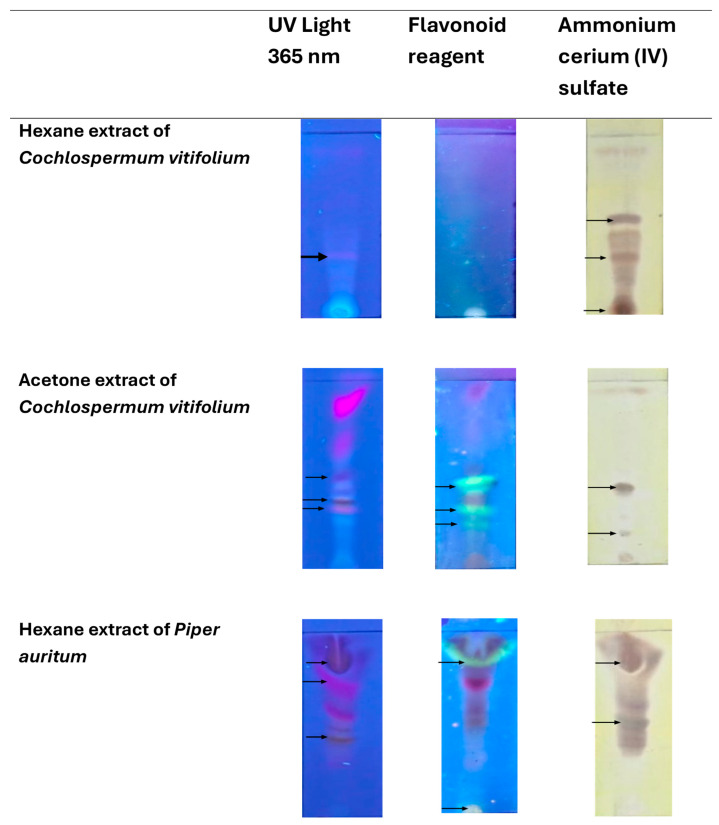
TLC visualization of hexane and acetone extracts of *Cochlospermum vitifolium* and *Piper auritum*. Chromatograms were observed under UV light at 365 nm and after derivatization with a flavonoid-specific reagent and ammonium cerium(IV) sulfate. The arrows indicate the fluorescent bands from orange-yellow to greenish-white, which suggested compounds similar to flavonoids, while dark brown bands after derivatization were consistent with the presence of terpenoid-type constituents.

TLC was used as an exploratory tool to compare the chemical profiles of hexane and acetone extracts of *S. mexicanum* and *E. heterophyllum* (Figure 11), and *C. vitifolium* and *Piper auritum* (Figure 12). Under UV irradiation at 365 nm, both extracts showed the presence of multiple fluorescent bands, indicative of aromatic and conjugated compounds, with visible differences in the intensity and distribution of the signals between species and solvents.

After development with a flavonoid reagent, orange-yellow and greenish-white bands were observed, particularly in the acetone extracts, suggesting a greater relative abundance of phenolic compounds of medium polarity. In contrast, treatment with cerium ammonium sulfate (IV) allowed the visualization of dark brown spots, mainly in the hexane extracts, consistent with the presence of lipophilic compounds, such as terpenes and other nonpolar metabolites. Overall, the TLC profiles revealed clear qualitative differences between the evaluated extracts, based on both the plant species and the extraction solvent. Overall, the TLC profiles revealed clear qualitative differences between the evaluated extracts, based on both the plant species and the extraction solvent.

#### 2.3.2. Quantification of Total Phenolic Content

The quantification of total phenolic content was performed for the medium- and high-polarity extracts, as phenolic compounds are widely recognized for their contribution to antiproliferative, pro-oxidant, and cytotoxic effects in cancer cells. This approach enabled a broader comparison of the chemical features that may underlie the biological activity observed among extracts obtained with solvents of different polarities.

The total phenolic content of the extracts was determined using the Folin–Ciocalteu method and expressed as milligrams of gallic acid equivalents per gram of extract (mg GAE/g). The results revealed marked differences among the analyzed plant species as well as between the solvents employed, reflecting variability in phenolic enrichment across extracts (Table 6).

The acetonic extract of *C. vitifolium* (CA) exhibited the highest concentration of phenolic compounds (231.19 ± 2.00 mg GAE/g), followed by the acetonic and methanolic extracts of *S. mexicanum* (SA and SM), with 205.98 ± 2.87 and 180.06 ± 1.55, respectively. In contrast, the methanolic and acetonic extracts of *E. heterophyllum* (EM, EA) and *P. auritum* (PM), as well as the methanolic extract of *C. vitifolium* (CM), showed substantially lower concentrations, ranging from 33.78 to 56.51 mg GAE/g.

In general, the extracts obtained with acetone showed higher phenolic concentrations compared to those obtained with methanol, suggesting that compounds of intermediate polarity predominate in the extract composition of these species. This result is consistent with previous reports indicating that solvents of intermediate polarity favor the recovery of these metabolites [19]. The difference was particularly notable in *C. vitifolium*, where the acetonic extract (CA) exhibited a significantly higher yield than its methanolic counterpart (CM).

An exception was *P. auritum*, whose methanolic extract (PM) showed a better yield than the acetonic one (PA). This finding suggests that the dominant phenolic fraction in *P. auritum* consists of more polar compounds, which are better solubilized in methanol. Overall, these results confirm that both plant species and solvent polarity are determining factors in the efficiency of polyphenol extraction.

The high total polyphenol content observed in *S. mexicanum* and *C. vitifolium* has important biological implications. The elevated phenolic content in these extracts could be directly associated with their ethnomedicinal uses and previously reported anti-inflammatory properties [20,21,22], as the activity of these compounds is largely linked to their antioxidant capacity.

#### 2.3.3. Chemical Profiling by GC–MS

Following the cytotoxic and antiproliferative evaluation, a complementary chemical analysis was performed to better understand the main classes of compounds potentially associated with the observed biological effects. While the hexane extract of *S. mexicanum* exhibited the highest cytotoxic potency and was therefore selected for GC-MS profiling, several acetone and methanolic extracts also showed relevant cytotoxic activity and, in some cases, favorable selectivity indices against cancer cell lines.

**Figure 13:**
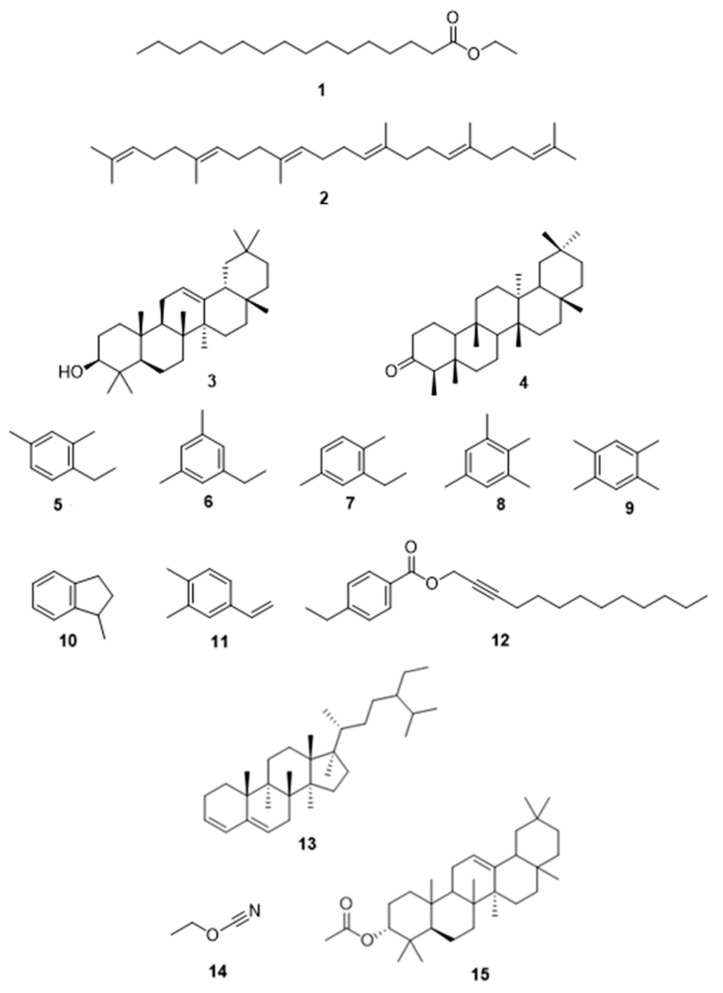
Chemical structures of compounds **1**–**15** identified in the hexane extract of *Semialarium mexicanum*. Structures were drawn using ChemDraw^®^ software (20.1.1) based on compounds tentatively identified by GC–MS analysis.

Among the samples evaluated, the hexane extract of *S. mexicanum* presented the most consistent and selective cytotoxic profile across all cervical cancer cell lines analyzed. Consequently, GC-MS analysis was applied as a preliminary chemical screening to explore its compositional profile and tentatively identify the main components that could be associated with its reproducible and selective anticancer effects (Figures S1–S3, Supplementary Materials). The chemical structures of these compounds were determined by analyzing their fragmentation patterns and comparing them with the NIST data library. The compounds identified were squalene (**2**) (11.43%), friedelan-3-one (**4**) (5.73%), *β*-amyrone (**3**) (1.70%) and ethyl palmitate (**1**) (0.50%) (Table 7(a) and Figure 11). Since not all constituents were detected in the initial analysis, the extract was fractionated for analysis by GC-MS. The separation produced two distinct fractions: first fraction the compounds identified were 1,2,4,5-tetramethylbenzene (**9**) (23.90%), 1,2,3,5-tetramethylbenzene (**8**) (7.25%), 4-ethenyl-1,2-dimethylbenzene (**11**) (4.01%),1-ethyl-3,5-dimethylbenzene (**6**) (3.72%), 1-ethyl-2,4-dimethylbenzene (**5**) (3.05%), stigmasta-3,5-diene (**13**) (2.23%), tridec-2-yn-1-yl 4-ethylbenzoate (**12**) (1.80%), 1-methylindane (**10**) (1.55%) and 2-ethyl-1,4-dimethylbenzene (**7**) (1.51%) (Table 7(b) and Figure 11), and second fraction contained 12-oleanen-3-yl acetate, (3.alpha.) (**15**) (3.77%) and ethyl cyanate (**14**) (2.29%) (Table 7(c) and Figure 13). It is important to mention that some components present in the extract and the fractions were not identified by this analysis.

## 3. Discussion

In this study, we demonstrate that hexane, acetone, and methanolic extracts of *S. mexicanum* exhibit pronounced selective cytotoxicity toward human cervical cancer cells, with SI greater than 5 in at least one HeLa cell line, indicating a marked preference for tumor cells over non-cancerous fibroblasts (HFF-1 and HUF). In contrast, extracts of *E. heterophyllum* and showed moderate SI values (3–4.5), reflecting cytotoxicity with a less discriminatory targeting of malignant cells. These findings highlight the importance of evaluating not only cytotoxic potency but also the therapeutic window: the ability to affect cancer cells without affecting healthy tissue, a key factor in the development of anticancer drugs and the determination of their safety.

Conventional chemotherapeutic agents, such as paclitaxel, maintain high potency but often lack sufficient selectivity, leading to dose-limiting toxicities in healthy tissues. In our model, paclitaxel exhibited SI values of 4.18 and 4.82 in the HeLa-HaCaT and HeLa-HUF comparisons, respectively, suggesting good, though not exceptional, selectivity. In contrast, *S. mexicanum* extracts achieved higher IS values even when effective concentrations exceeded those required for paclitaxel cytotoxicity, indicating a potentially wider safety margin—an essential characteristic for compounds derived from traditional medicinal plants that can reduce unwanted effects. Studies analyzing natural products have also highlighted the value of extracts that inhibit tumor cell viability with minimal effects on normal cells, reinforcing the therapeutic promise of plant-derived agents with selective profiles [23,24,25,26,27].

In all non-cancerous cell models analyzed (HFF-1, HUF, HaCaT, HEK293), consistently higher *IC*_50_ values were observed compared to cancer cells, suggesting lower sensitivity and supporting the safety profile of the extracts. Among these, HUF and HFF-1 fibroblasts were the least susceptible, followed by HaCaT keratinocytes and HEK293 cells. The inclusion of multiple non-cancerous cell lines, particularly HUFs as a direct comparator with HeLa cervical cancer cells, reinforces the evidence of a preference for malignant over normal phenotypes [28,29,30,31,32].

The selective cytotoxicity of plant extracts has been documented in various studies with medicinal plants. For example, ethyl acetate fractions from *Citrullus colocynthis* demonstrated potent inhibition of the viability of pancreatic and cutaneous cancer cells, with minimal effects on normal BJ-1 cells, suggesting significant selectivity [33,34].

The selective cytotoxicity observed for *S. mexicanum*, particularly in the hexane extract, may be related to its chemical composition. GC–MS analysis of the hexanic stem extract revealed a complex mixture mainly composed of alkylated aromatic hydrocarbons and triterpenoid- and sterol-related compounds, including benzene derivatives, squalene, β-amyrone, stigmasterol-related structures, and friedelan-3-one. Although these compounds were tentatively identified and not evaluated individually in this study, several structurally related triterpenoids and sterols have previously been associated with antiproliferative and pro-apoptotic effects in cancer cell models, providing contextual support for the observed biological activity of the extract as a whole [35,36,37,38,39]. Notably, squalene and pentacyclic triterpenoids such as amyrins and friedelane derivatives have been reported to modulate oxidative stress, membrane integrity, and apoptosis-related signaling pathways in tumor cells, while exhibiting relatively low toxicity toward non-malignant cells. The enrichment of such non-polar bioactive constituents in the hexane extract is consistent with its higher cytotoxic potency and selectivity compared to more polar extracts [40,41,42].

In contrast, acetonic and methanolic extracts of *S. mexicanum* showed a high content of total phenolic compounds, suggesting that phenolics may also contribute to the antiproliferative effects observed for these fractions. Phenolic compounds are widely recognized for their ability to interfere with cell cycle progression, induce oxidative imbalance, and activate programmed cell death pathways in cancer cells, while often displaying lower cytotoxicity toward normal cells [43,44,45,46].

Meanwhile, the cell cycle data revealed that all extracts significantly increased the sub-G1 population in HeLa cells, an indicator of DNA fragmentation associated with apoptotic processes, with the hexane extract from S. mexicanum producing the most pronounced effect [47].

This observation is consistent with reports indicating that plant extracts induce cell cycle disruption and apoptosis in cancer cells, a common mechanism attributed to phytochemicals such as flavonoids, alkaloids, and phenolic compounds that modulate cell death pathways. Furthermore, DAPI staining provided visual evidence of nuclear condensation and fragmentation in treated HeLa cells, further supporting the antiproliferative and cytotoxic effects of the extracts [48]. While the exact molecular mechanisms remain unclear, these combined findings suggest that the action of the extracts may involve programmed cell death pathways, a desirable attribute in anticancer agents [49,50].

Although the present study did not directly evaluate apoptosis-related signaling proteins such as caspases or Bcl-2 family members, the interpretation of programmed cell death involvement is based on converging evidence from cell cycle alterations and characteristic nuclear morphological changes. Further studies addressing specific apoptotic markers will be required to fully elucidate the molecular mechanisms underlying these effects.

The GC–MS analysis of the hexane extract of *S. mexicanum* revealed a predominance of simple alkylated benzene derivatives. Although alkylated benzenes have been sporadically reported as constituents of certain plant extracts and essential oils, particularly in species exposed to environmental stress or anthropogenic influence, their occurrence as major components in plant-derived extracts is relatively uncommon. Moreover, volatile compounds of synthetic or artificial origin have been documented as common contaminants in plant extracts and essential oils, which can be inadvertently introduced during sample preparation or instrumental analysis [51].

On the other hand, related aromatic compounds, such as alkenylbenzenes, have been identified as natural constituents in essential oils and volatile fractions of certain herbs and plant species, indicating that substituted benzene derivatives can occasionally occur in plant metabolomes, although generally at low concentrations and with limited quantitative data [52].

Therefore, while the presence of these compounds should be interpreted with caution, the GC–MS analysis provides a preliminary chemical profile that is valuable for guiding subsequent studies. The reliability of GC–MS library-based identification does not rely exclusively on numerical match factors but also depends on prior probability, chromatographic behavior, and expert interpretation, particularly in complex matrices such as plant extracts, as discussed by Stein et al. [53]. Importantly, the combined presence of lipophilic triterpenes and phenolic constituents in *S. mexicanum* extracts provides a plausible biochemical basis for the preferential cytotoxicity toward cancer cells observed in this study. Overall, despite the tentative nature of some GC–MS identifications, the chemical diversity revealed supports the observed biological activity and highlights the extract as a promising candidate for further phytochemical characterization and mechanistic investigations.

## 4. Materials and Methods

### 4.1. Collection of Plant Materials

The plant material was collected in various locations: *P. auritum* (commonly known as “Hoja Santa”) in Álvaro Leonel, La Joya, Morelos (18°53′30.0″ N, 99°07′15.8″ W); *E. heterophyllum* (“Hierba del Sapo”) in the municipality of Ixtapan de la Sal, State of Mexico (18°53′25″ N, 99°40′24″ W); *S. mexicanum* (“Cancerina”) in Los Naranjos, Guerrero (18°24′11.9″ N, 99°31′59.5″ W); and *C. vitifolium* (“Panicua”) in the Lomas del Carril neighborhood of Temixco, Morelos (18°51′38.646″ N, 99°13′55.480″ W). Once the specimen was taxonomically authenticated, it was deposited in the HUMO-UAEM Herbarium. Drying was performed at 50 °C under continuous air circulation for 72 h, until constant weight was obtained, and identical drying conditions were used for all plant species to ensure experimental consistency.

### 4.2. Plant Material Extraction

The plant material of each species was finely powdered using an IKA^®^ electric mill (Basic Microfine Motor MF 10; Wilmington, NC, USA) and subjected to successive maceration extraction using organic solvents of increasing polarity (hexane, acetone, and methanol). Briefly, 150 g of plant powder were initially macerated with 1 L of hexane in a 1 L amber glass flask for 72 h at room temperature with occasional manual agitation. After filtration through Whatman No. 16 filter paper, the hexane extract was recovered and concentrated to dryness under reduced pressure using a rotary evaporator (R-114; Büchi Labortechnik AG, Flawil, Switzerland) at 50 °C and 300 mbar. The same procedure was subsequently applied using acetone (40 °C, 250 mbar) and methanol (60 °C, 150 mbar).

The residual plant material was then air-dried and subsequently extracted with 1 L of acetone under the same maceration conditions. Following filtration and solvent removal, a final extraction step was performed using methanol. Each extraction step was conducted sequentially on the same plant material residue, and the entire procedure was performed in triplicate. The resulting dried extracts were stored at −20 °C until further analysis.

Preliminary chromatographic analysis was carried out using thin-layer chromatography (TLC) on silica gel 60 F_254_ plates (Merck, Darmstadt, Germany), and the hexane extract was fractionated by column chromatography (CC) on silica gel using 70–230 mesh silica gel (high purity grade; Sigma-Aldrich, Burlington, MA, USA) as the stationary phase. For TLC, the plates were developed with two different reagents: 2-aminoethyl diphenylborinate (NP-PEG) as a specific reagent for flavonoid detection, and ammonium cerium (IV) sulfate as a universal developer. Visualization of the separated compounds was performed under ultraviolet light at wavelengths of 254 and 365 nm using a portable UV lamp (UVP UVGL-25).

### 4.3. Cell Culture and Cytotoxicity Assay

The cytotoxic activity of the plant extracts was evaluated using the crystal violet assay in a panel of human cancer and non-malignant cell lines, including human cancer cell lines HeLa (cervical adenocarcinoma), SiHa (cervical squamous cell carcinoma), HepG2 (hepatocellular carcinoma), PC3 (prostate adenocarcinoma), H1299 (non-small cell lung carcinoma), and MCF-7 (breast adenocarcinoma) were used in this study. Non-tumoral models included human uterine fibroblasts (HUFs, primary cells), human foreskin fibroblasts (HFF-1), human embryonic kidney cells (HEK293), and human keratinocytes (HaCaT).

All cell lines were obtained from the American Type Culture Collection (ATCC, Manassas, VA, USA). HaCaT and primary HUF cells were kindly provided by recognized academic laboratories and were originally sourced from ATCC. HaCaT cells, which were kindly provided by Dr. Alexandre Toshirrico Cardoso Taketa, Full-Time Research Professor (Titular B) at the Centro de Investigación en Biotecnología, Universidad Autónoma del Estado de Morelos (UAEM), and primary human uterine fibroblasts (HUFs), which were kindly donated by Dr. Heriberto Abraham Valencia González, researcher at the Instituto Nacional de Cancerología (INCan). All cells were maintained at low passage numbers and cultured under standardized aseptic conditions. Cell morphology, growth behavior, and biological consistency were routinely monitored, and all cultures were periodically tested for mycoplasma contamination using standard detection methods.

Cells were cultured at 37 °C in a humidified atmosphere with 5% CO_2_. Culture media and fetal bovine serum (FBS) were purchased from Invitrogen (Thermo Fisher Scientific, Waltham, MA, USA) and supplemented with 10% FBS and 1% penicillin–streptomycin. Specifically, HeLa and HaCaT cells were maintained in DMEM, MCF-7, SiHa and HEK293 cells in Eagle’s Minimum Essential Medium (EMEM), PC3 in RPMI, and HUF and HepG2 cells in DMEM/F-12. The remaining cell lines were cultured according to ATCC-recommended conditions.

For cytotoxicity evaluation, cells were seeded at a density of 5000 cells per well in 96-well plates and allowed to adhere for 48 h. Cells were then treated with increasing concentrations of the plant extracts (200, 100, 50, 25, and 12.5 µg/mL) for 24 h. Paclitaxel was used as a positive control, while dimethyl sulfoxide (DMSO) served as the vehicle control, with a final concentration not exceeding 0.1% (*v*/*v*).

After treatment, cells were gently washed with phosphate-buffered saline (PBS), fixed with 4% paraformaldehyde and stained with 0.5% (*w*/*v*) crystal violet solution for 1 h. Excess dye was removed by thorough washing with distilled water, and plates were air-dried. The bound dye was solubilized using SDS 10%, and absorbance was measured at 570 nm using an automated ELISA reader (Promega, Madison, WI, USA). Cell viability was expressed as a percentage relative to untreated control cells.

#### Data Analysis

Concentration– response curves were generated, and *IC*_50_ values were calculated by nonlinear regression analysis using GraphPad PRISM software (version 8.0; GraphPad Software, San Diego, CA, USA). All experiments were performed in triplicate and results are presented as mean ± SD. Statistical analysis was carried out using one-way ANOVA followed by Dunnett’s multiple comparisons test. HeLa cells were used as the reference group for comparisons with normal cell lines. A value of *p* < 0.05 was considered statistically significant.

### 4.4. Cell Cycle Analysis

HeLa cells (1.5 × 10^5^ cells/well) were seeded in 6-well plates. Exponentially growing cells were exposed for 24 h to the hexane, acetone, and methanol extracts of *S. mexicanum*, *E. heterophyllum*, and *C. vitifolium* at a concentration of 20 µg/mL, selected as a standardized working concentration, as most *IC*_50_ values of the active extracts in HeLa cells fell within a comparable range s. Paclitaxel (20 and 100 nM) was included as a positive control for G_2_/M phase arrest.

Following treatment, cells were trypsinized, collected as single-cell suspensions, centrifuged, and fixed overnight in cold 70% ethanol at −20 °C. Fixed cells were then treated with RNase A (0.01 M; Sigma-Aldrich) and stained with propidium iodide (PI; 7.5 µg/mL; Invitrogen) for 30 min in the dark. RNase treatment was included to eliminate RNA interference, allowing PI to bind specifically to cellular DNA.

Cell cycle distribution (G_0_/G_1_, S, and G_2_/M phases) was analyzed using a flow cytometer (FACS Lyric™, Becton Dickinson, Franklin Lakes, NJ, USA), acquiring 10,000 events per sample. All experiments were performed in triplicate and repeated in three independent experiments. Flow cytometry data were analyzed using FlowJo software v10.8.1 ((BD Life Sciences, Franklin Lakes, NJ, USA) to generate DNA content histograms and quantify the percentage of cells in each cell cycle phase.

### 4.5. Nuclear Morphology Analysis by DAPI Staining

HeLa cells were seeded at a density of 1.25 × 10^5^ cells per well in 6-well plates and allowed to attach overnight. For nuclear morphology DAPI analysis, HeLa cells were treated for 24 h with the extracts at 20 µg/mL, selected as a standardized concentration to ensure consistency with cell cycle experiments. Paclitaxel (20 and 100 nM) was used as a positive control for apoptosis induction, while untreated cells served as the negative control.

After treatment, cells were washed twice with PBS and fixed with 4% paraformaldehyde for 15 min at room temperature. Fixed cells were subsequently permeabilized with 0.1% Triton X-100 in PBS for 5 min, washed with PBS, and stained with 4′,6-diamidino-2-phenylindole (DAPI; 1 µg/mL) for 10 min in the dark.

Nuclear morphology was examined using a fluorescence microscope equipped with a DAPI filter. Apoptotic features such as chromatin condensation, nuclear fragmentation, and apoptotic body formation were qualitatively assessed. Images were captured from randomly selected fields under identical acquisition settings. Experiments were performed in triplicate and repeated in three independent assays.

### 4.6. Quantification of Total Phenolic Content

The total polyphenol content (TPC) was quantified using the Folin–Ciocalteu method [54]. Briefly, an aliquot of 28 μL of extract, dissolved in 8:2 H_2_O–MeOH (0.9 mg/mL), was mixed with 42 μL of Folin–Ciocalteu reagent (1 N). The mixture was kept on an orbital shaker at 80 ± 1 rpm and incubated at room temperature in the dark for 5 min. Then, 42 μL of Na_2_CO_3_ solution (20% *w*/*v*) and 168 μL of distilled water were added, followed by incubation under the same conditions for 30 min. Samples were immediately read against a blank on a Multiscan Go spectrophotometer at 760 nm. The TPC was calculated by comparison with a gallic acid standard curve (5–100 μg/mL; y = 0.0094x + 0.0803; R^2^ = 0.9999). Results are expressed as mg of gallic acid equivalents per g of dry extract (mg GAE/g extract). All determinations were performed in triplicate *(n* = 3) and are reported as mean ± standard deviation.

### 4.7. GC-MS Analysis

The hexane extract of *S. mexicanum* was analyzed at the Mass Spectrometry Laboratory of the Institute of Chemistry, National Autonomous University of Mexico. The analysis was performed using gas chromatography-mass spectrometry (GC-MS) with an Agilent Technologies 7890A gas chromatograph (Agilent Technologies, Santa Clara, CA, USA) coupled to a Jeol GC Mate II mass spectrometer. Separation was carried out on a 30 m long, 0.32 mm internal diameter, 0.25 µm film thickness NP-5 capillary column (5% diphenyl/95% dimethylpolysilox-ane). Helium was used as the carrier gas at a constant flow rate of 1 mL/min. The in-jector temperature was maintained at 300 °C, and 1 µL of sample was injected in split mode (50:1). The samples were dissolved in hexane at a concentration of 1 mg/mL prior to injection. The oven temperature program started at 40 °C (maintained for 1 min) and increased to 300 °C at a rate of 8 °C/min. For detection, the mass spectrometer operated in electron impact ionization mode at 70 eV, recording a mass range of *m*/*z* 10 to 600 with a scan time of 1.3 s. The identification of the substances was made by comparison with data library of the National Institute of Standards and Technology (NIST).

## 5. Conclusions

This study provides a comprehensive in vitro evaluation of the selective cytotoxic and antiproliferative effects of extracts from four Mexican medicinal plants (*S. mexicanum, E. heterophyllum*, *P. auritum*, and *C. vitifolium*) across a diverse panel of human cancer and non-cancerous cell lines, emphasizing selectivity as a decisive parameter in anticancer screening. Among the species analyzed, *S. mexicanum* emerged as the most promising candidate, exhibiting pronounced cytotoxic potency and preferential activity toward HeLa cervical cancer cells, particularly in its hexane extract, with selectivity indices exceeding those observed for paclitaxel when compared with primary human uterine fibroblasts (HUFs).

Hexane extracts of *S. mexicanum* and *E. heterophyllum* were generally more active than their corresponding polar extracts, whereas *P. auritum* exhibited limited cytotoxicity and *C. vitifolium* displayed moderate, solvent-dependent effects. The use of physiologically relevant non-tumoral models, especially HUF cells as direct comparators for cervical cancer cell lines, strengthens the assessment of tumor selectivity.

Furthermore, the cell cycle alterations and nuclear morphological changes observed in HeLa cells support an antiproliferative response consistent with the activation of cell death pathways. When integrated with the chemical profile data, these biological effects suggest that the enrichment of lipophilic triterpenoids and phenolic components in *S. mexicanum* extracts could contribute to their selective cytotoxic profile.

Although confined to in vitro systems, the present study establishes a toxicologically informed screening strategy for medicinal plant extracts and supports the value of selectivity-guided evaluation as a critical step preceding chemical isolation, mechanistic elucidation, and more advanced safety assessments.

## Figures and Tables

**Table 1:** Taxonomic information, geographic origin, and collection parameters of the plant species under study.

Name	Family	Voucher Specimen Number	Plant Organ Used	Collection Date	Collection Site
*Cochlospermum* *vitifolium*	Bixaceae	28,904	trunk and stem	4 February 2024	Lomas del Carril neighborhood of Temixco, Morelos
*Eryngium* *heterophyllum*	Apiaceae	29,010	whole plant without roots	6 February 2024	State Of Mexico
*Piper auritum*	Piperaceae	28,935	aerial plant parts	7 February 2024	Álvaro Leonel, La Joya, Morelos
*Semialarium* *mexicanum*	Celastraceae	32,942	trunk and stem	15 February 2024	Los Naranjos, Guerrero

**Table 2:** Yields of hexanic, acetone and methanolic extracts of *C. vitifolium*, *E. heterophyllum*, *P. auritum* and *S. mexicanum*.

Plant Species	Hexane		Acetone		Methanol	
	Extract (g)	Extract Yields (%)	Extract (g)	Extract Yields (%)	Extract (g)	Extract Yields (%)
*Cochlospermum vitifolium*	0.73	0.49	1.92	1.28	12.08	9.39
*Eryngium heterophyllum*	1.92	1.28	3.36	2.24	14.71	9.81
*Piper auritum*	3.21	2.14	24.03	16.02	24.30	16.2
*Semialarium* *mexicanum*	1.71	1.14	6.90	4.60	14.29	9.53

**Table 3:** *IC*_50_ values (mean ± SD) of plant extracts and paclitaxel in human cancer cell lines. *IC*_50_ values for plant extracts are expressed in µg/mL, whereas paclitaxel values are expressed in nM. Data represent the mean ± SD of three independent experiments performed in triplicate.

Plant Species	Extract		Cancerous Cell Lines				
		HeLa	SiHa	HepG2	PC-3	H1299	MCF-7
*Eryngium heterophyllum*	Hexane	24.58 ± 4.0	20.4 ± 2.4	34.0 ± 4.5	28.3 ± 4.1	29.5 ± 7.5	32.7 ± 3.7
	Acetone	29.35 ± 3.9	29.4 ± 3.5	32.6 ± 3.9	40.2 ± 6.9	45.7 ± 2.5	49.3 ± 3.5
	Methanolic	24.5 ± 3.6	27.45 ± 3.7	40.4 ± 3.7	29.8 ± 5.7	65.7 ± 7.2	59.3 ± 3.7
*Cochlospermun vitifolium*	Hexane	59.5 ± 2.59	55.78 ± 4.0	82.6 ± 2.4	68.4 ± 2.6	78.5 ± 3.2	58.3 ± 6.1
	Acetone	39.6 ± 4.1	42.3 ± 5	45.5 ± 5.1	32.1 ± 3.7	62.1 ± 4.5	42.3 ± 2.4
	Methanolic	19.19± 3.3	20.45 ± 2.5	20.4 ± 2.3	30.1 ± 3.2	35.1 ± 5.7	30.1 ± 6.2
*Semialarium* *mexicanum*	Hexane	15.9 ± 1.8	17.16 ± 2.8	24.7.± 2.7	23.8 ± 4.5	38.02 ± 10.1	26.48 ± 6.4
	Acetone	19.3 ± 2.8	21.78 ± 3.2	37.0 ± 2.7	29.5 ± 5.5	45 ± 4.3	29.4 ± 3.3
	Methanolic	21.2 ± 3.1	23.5 ± 2.7	24.4 ± 3.7	51.2 ± 4.8	41 ± 7.2	66.8 ± 6.3
*Piper auritum*	Hexane	220.2 ± 4.6	240.7 ± 7.1	199.5 ± 2.9	201.4 ± 2.4	245 ± 20.7	289.9 ± 14.5
	Acetone	230.6 ± 2.0	230.6 ± 2.0	212.7 ± 3.3	251.7 ± 2.2	277.1 ± 27.4	256.2 ± 16.9
	Methanolic	50.7 ± 5.1	75.5 ± 5.1	160.4 ± 2.2	78.5 ± 2.7	121.4 ± 14.5	135.78 ± 12.8
Paclitaxel nM		15.7 ± 1.6	25.7 ± 2.2	68.7 ± 17.9	70.56 ±4.9	70.79 ± 2.7	108.52 ± 7.2

**Table 4:** *IC*_50_ values (mean ± SD) of the most cytotoxic extracts from *Semialarium mexicanum* and Eryngium heterophyllum in non-cancerous cell lines.

Plant Species	Sample	Human Foreskin Fibroblasts	Primary Uterine Fibroblast Cells	Immortalized Human Keratinocytes	Embryonic Kidney Immortalized Cell Line
		HFF-1	HUF	HaCat	HeK-293
*Semialarium* *mexicanum*	Hexane	79.46 ± 5.5	89.71 ± 4.3	53.53. ± 6.2	40.4 ± 5.5
	Acetone	114.2 ± 6.1	119.2 ± 4.7	61.59 ± 6.9	49.17 ± 5.3
	Methanolic	113.5 ± 5.4	122.4 ± 4.2	93.79 ± 4.18	89.2 ± 2.7
*Eryngium* *heterophyllum*	Hexane	117.3 ± 5.9	75.8 ± 5.1	58.85 ± 6.0	41.67 ± 6.7
	Acetone	* ND	113.9 ± 3.7	104.0 ± 3.2	75.41 ± 6.1
	Methanolic	108.5 ± 4.3	88.6 ± 5.0	75.25 ± 6.2	70.2 ± 4.4
Paclitaxel (nM)		* ND	75.7 ± 5.0	65.79 ± 5.0	37.8 ± 2.5

* ND: not determined.

**Table 5:** SI values of *Eryngium heterophyllum* and *Semialarium mexicanum* extracts in HeLa and SiHa cells calculated using non-cancerous cell lines (HFF-1, HUF and HaCat as reference).

Plant Species	Sample	HeLa			SiHa Cells		
		HFF-1	HaCat	HUF	HFF-1	HaCat	HUF
*Eryngium* *heterophyllum*	Hexane	4.77	1.27	3.05	5.75	2.88	3.75
	Acetone	* ND	3.5	3.87	* ND	3.5	3.8
	Methanolic	4.42	3.07	3.6	3.9	2.74	3.22
*Semialarium* *mexicanum*	Hexane	4.99	3.36	5.64	4.6	3.11	5.22
	Acetone	5.92	3.19	6.17	5.24	2.82	5.48
	Methanolic	5.35	4.42	5.77	4.8	3.99	5.2
	Paclitaxel	* ND	4.18	4.82	* ND	2.55	2.94

* ND: not determined.

**Table 6:** Total Phenolic Content (mg GAE/g extract) of Methanolic and Acetonic Extracts.

Extract	Total Phenolic Content (mg GAE/g Extract)
SA	205.98 ± 2.87
SM	180.06 ± 1.55
EA	40.79 ± 0.82
EM	33.78 ± 1.26
CA	231.19 ± 2.00
CM	56.51 ± 0.46
PA	45.68 ± 1.02
PM	54.82 ± 1.27

*Semialarium mexicanum* methanolic extract = SM; *Semialarium mexicanum* acetonic extract = SA; *Cochlospermum vitifolium* methanolic extract = CM; *Cochlospermum vitifolium* acetonic extract = CA; *Eryngium heterophyllum* methanolic extract = EM; *Eryngium heterophyllum* acetonic extract = EA; *Piper auritum* methanolic extract = PM; *Piper auritum* acetonic extract = PA.

**Table 7:** Chemical profile of the hexane extract of *Semialarium mexicanum* obtained by GC–MS analysis.

	No	Name	* RT	Area %	Match %
a	**1**	Ethyl palmitate	19.47	0.50	78.8
	**2**	Squalene	27.72	11.43	95.6
	**3**	*β*-amyrone	31.36	1.70	75.7
	**4**	Friedelan-3-one	32.79	5.73	89.6
b	**5**	1-ethyl-2,4-dimethylbenzene	5.95	3.05	88.3
	**6**	1-ethyl-3,5-dimethylbenzene	6.06	3.72	94.5
	**7**	2-ethyl-1,4-dimethylbenzene	6.41	1.51	92.4
	**8**	1,2,3,5-tetramethylbenzene	6.58	7.25	95.5
	**9**	1,2,4,5-tetramethylbenzene	6.65	23.90	97.4
	**10**	1-Methylindane	6.96	1.55	77.1
	**11**	4-ethenyl-1,2-dimethylbenzene-	7.13	4.01	90.5
	**12**	Tridec-2-yn-1-yl 4-ethylbenzoate	8.03	1.80	85.7
	**13**	Stigmasta-3,5-diene	29.69	2.23	76.7
c	**14**	Ethyl cyanate	10.94	2.29	84.9
	**15**	12-Oleanen-3-yl acetate, (3.alpha.)	29.07	3.77	79.6

RT = Retention Time *.

## Data Availability

The original contributions presented in this study are included in the article/Supplementary Materials. Further inquiries can be directed to the corresponding author.

## Supplementary Materials

The following supporting information can be downloaded at: https://www.mdpi.com/article/10.3390/molecules31030549/s1, Figure S1. GC-MS chromatogram of the hexane extract from *S. mexicanum* (ExtHexJd); Figure S2. GC-MS chromatogram of the first fraction of the hexane extract of *S. mexicanum* (He11s.d); Figure S3. GC-MS chromatogram of the second fraction of the hexane extract of *S. mexicanum* (He12s.d).
