# Supplementary material for: Selective Cytotoxic and Antiproliferative Effects of Extracts from Four Mexican Medicinal Plants in Human Cancer and Non-Cancerous Cell Lines
Source: Molecules. 2026 Feb 4;31(3):549. doi: 10.3390/molecules31030549 (PMC12899643; doi:10.3390/molecules31030549)
Supplement: Supplementary file 1 [file molecules-31-00549-s001.zip › molecules-4109195-supplementary.pdf]

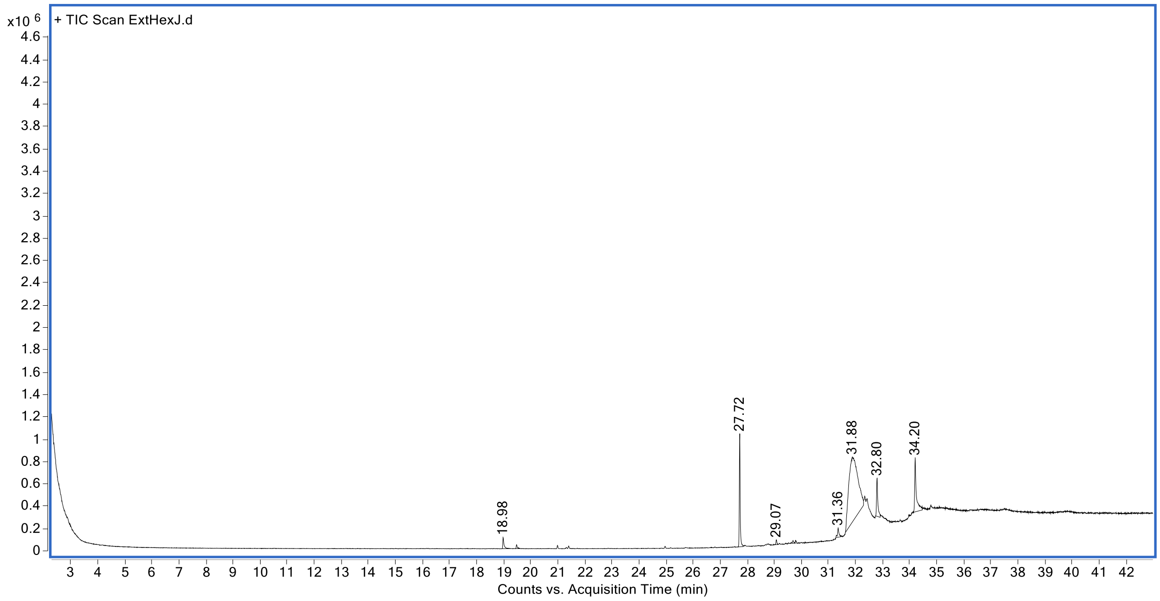

Figure S1. GC-MS chromatogram of the hexane extract from *S. mexicanum* (ExtHexJd).

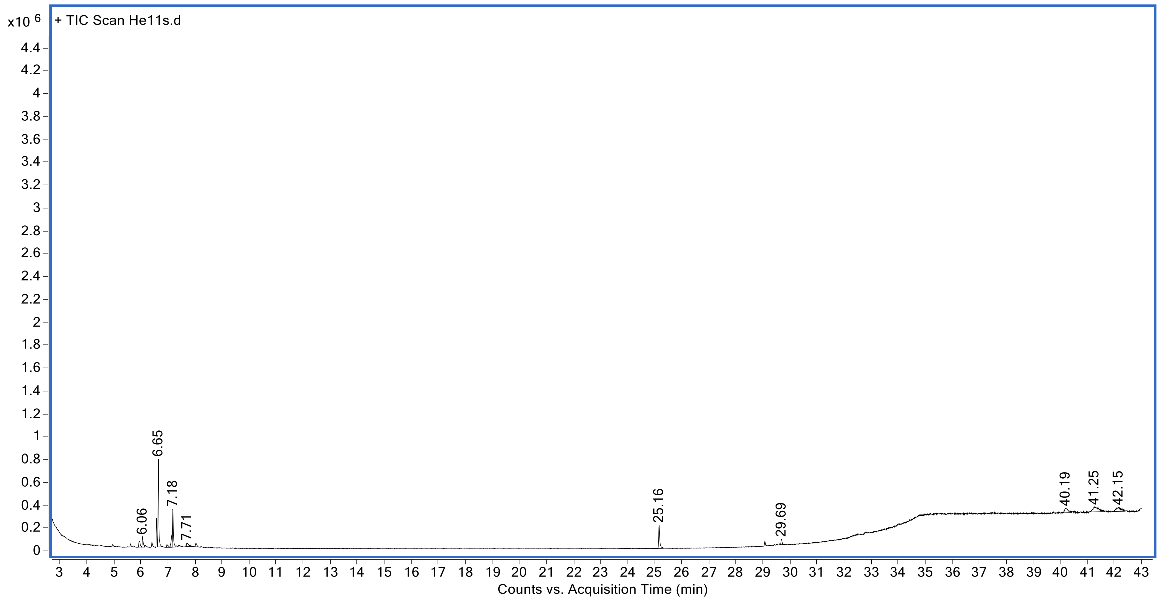

Figure S2. GC-MS chromatogram of the first fraction of the hexane extract of *S. mexicanum* (He11s.d)

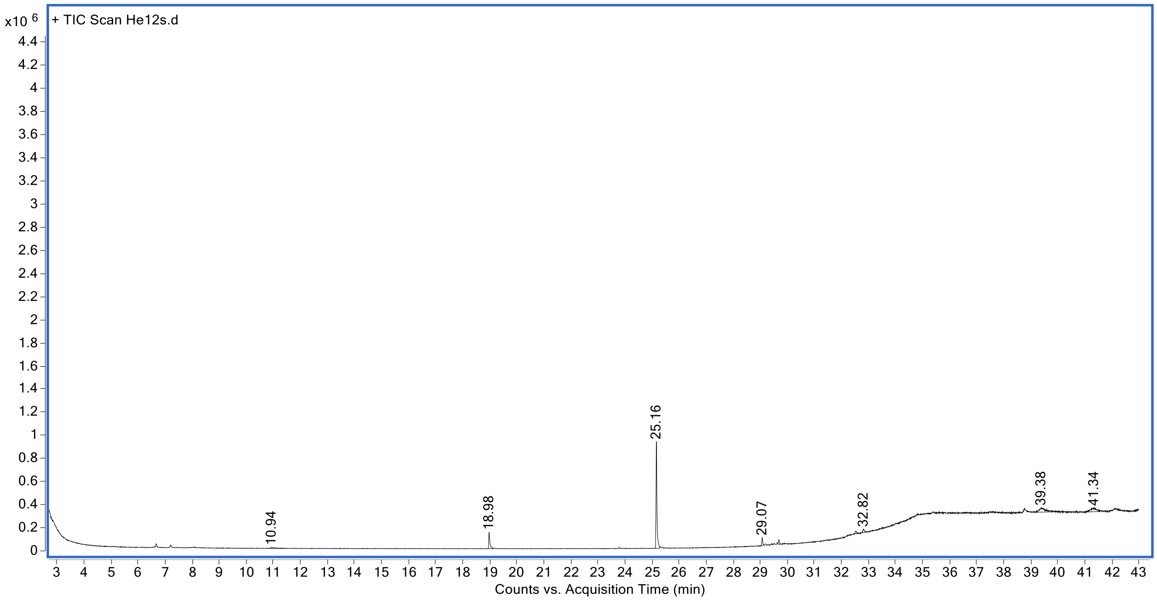

Figure S3. GC-MS chromatogram of the second fraction of the hexane extract of *S. mexicanum* (He12s.d).
